# IL-10-Producing B Cells Suppress Effector T Cells Activation and Promote Regulatory T Cells in Crystalline Silica-Induced Inflammatory Response In Vitro

**DOI:** 10.1155/2017/8415094

**Published:** 2017-08-02

**Authors:** Yiping Lu, Fangwei Liu, Chao Li, Ying Chen, Dong Weng, Jie Chen

**Affiliations:** ^1^Division of Pneumoconiosis, School of Public Health, China Medical University, Shenyang, China; ^2^Department of Respiratory Medicine, Shanghai Pulmonary Hospital, Tongji University School of Medicine, Shanghai, China

## Abstract

Long-term exposure to crystalline silica leads to silicosis, which is characterized by persistent lung inflammation and lung fibrosis. Multiple immune cells have been demonstrated to participate in crystalline silica-induced immune responses. Our previous study indicated that B10 could control lung inflammation through modulating the Th balance in experimental silicosis in mice. However, the regulatory mechanism of B10 on CD4^+^ T cells is still unclear. MACS-sorted CD19^+^ B cells from the three different groups were cultured with CD4^+^ T cells either with or without transwell insert plates to evaluate the effects of B10 on CD4^+^ T cells, including Teff and Treg. B10 was eliminated by anti-CD22 application in vivo. Flow cytometry was used to test the frequencies of CD4^+^ T cells, and the expressions of the related cytokines were detected by real-time PCR and CBA. Insufficient B10 elevated the levels of proinflammatory cytokines and promoted Th responses in a way independent upon cell-cell contact in the Teff and B cell coculture system. B10 could both increase Treg activity and enhance conversion of Teff into Treg. Our findings demonstrated that B10 could affect Th responses by the release of IL-10, enhancing Treg functions and converting Teff into Treg.

## 1. Introduction

Occupational exposure to particulates such as crystalline silica is a global cause of respiratory disease occurred in numerous industrial settings including mining, glass, drilling, and sawing [1]. Over the past decade, many efforts have been made to prevent the workers from exposure to crystalline silica; however, silicosis induced by crystalline silica exposure is still a global heavy burden [2–4]. Inhalation of crystalline silica leads to activation and recruitment of lymphocytes, resulting in lung inflammation and fibrosis [5, 6]. On account of impaired particle clearance, silicosis is irreversible and incurable, leading to sustaining lung inflammation [7]. Therefore, to explore its pathogenesis and regulatory mechanism is particularly important for effective treatment of silicosis.

Crystalline silica is first recognized by macrophages, and then T cells and B cells can be activated [8, 9]. In the past, B cells are known to produce antibodies and proinflammatory cytokines and present antigens to activate T cell-mediated immune responses [10]. However, a novel subset of B cells, regulatory B cells (Breg), has been found [11]. Breg exerts immunosuppressive functions in tumor, autoimmunity, infections, and inflammation [12–15]. Although the specific phenotypes of Breg are varied in different diseases, the secretion of IL-10 is a unique feature of Breg [16–19]. As a result, CD19^+^ IL-10^+^ Breg is also known as IL-10-producing B cells (B10) [20]. Much attention has been paid to the role of Breg on T cells. Researchers found that Breg could reduce Th1/Th17 responses and induce Treg [21–24].

Our previous studies demonstrated that CD4^+^ T helper (Th) cells played a crucial role in immune response in silicosis. CD4^+^CD25^−^ effector T cells (Teff), such as Th1, Th2, and Th17, took part in different stages of silicosis according to the murine studies [25–27]. CD4^+^CD25^+^ regulatory T cells (Treg) were inducible and made efforts to modulate the Th responses after crystalline silica exposure [28–30]. The immune homeostasis and the balance among different Th responses determined the progress of silicosis. We also found that B10 could modulate the progress of crystalline silica-induced lung inflammation and fibrosis by suppressing the Th1 response and promoting Treg function in mice, which was consistent with previous studies [31]. However, the actual role of B10 on CD4^+^ T cells in silicosis still needs further exploration.

To study the modulatory function of B10 on Teff and Treg, respectively, we designed a series of studies in vitro. Anti-CD22 mAb, which was reported to eliminate B10, was used to restrict the expansion of crystalline silica-induced B10 [31–33]. Crystalline silica particle was used to trigger the B10 expansion in mice. CD19^+^ B cells, CD4^+^CD25^−^ Teff, and CD4^+^CD25^+^ Treg were isolated from different groups of mice. A CD19^+^ B cell and Teff/Treg coculture system was set up in vitro. Our study demonstrated that B10 could suppress the levels of crystalline silica-activated proinflammatory cytokines in cocultured system in vitro. The suppressive function of B10 on Th responses was independent upon cell-cell contact. B10 could both affect Treg and promote the conversion of CD4^+^CD25^−^ Teff into Treg, which could subsequently suppress the Th responses.

## 2. Materials and Methods

### 2.1. Animals

Female C57BL/6 mice were purchased from SLAC Laboratory Animal Co. Ltd. (Shanghai, China) at 6–8 weeks of age. All mice were maintained in a specific pathogen-free conditions and fed on a standard mouse chow at an environmental temperature of 24 ± 1°C and a 12 h/12 h light/dark cycle with water available ad libitum. The animal study was approved by the Animal Care and Use Committee of China Medical University (CMU62043018), which complies with the National Institutes of Health Guide for the Care and Use of Laboratory Animals. The study was performed in accordance with the approved guidelines.

### 2.2. Crystalline Silica Exposure and B10 Depletion

Natural crystalline silica particles (Min-U-Sil 5 ground silica; size distribution: 97% < 5 *μ*m diameter, 80% < 3 *μ*m diameter; median diameter 1.4 *μ*m) were obtained from the U.S. Silica Company (Frederick, MD). Crystalline silica particles were boiled in 1 N HCl to remove endotoxins. Before use, the suspension was autoclaved and then sonicated.

Mice were randomly distributed into three groups: the control group, the silica group, and the silica&anti-CD22 group, containing fifteen animals each. All mice were anesthetized with intraperitoneal injection of 2% pentobarbital sodium (45 mg/kg body weight). The silica group and the silica&anti-CD22 group mice received 3 mg/50 *μ*l crystalline silica suspension intratracheally to induce experimental silicosis. The control group mice received 50 *μ*l sterile saline.

For continuing depletion of CD19^+^ IL-10^+^ regulatory B cell, the silica&anti-CD22 group mice received 300 *μ*g anti-CD22 mAb (KH2014176, F239, Sangon Biotech, Shanghai, China) one day before the crystalline silica exposure and six days after crystalline silica exposure, respectively. All mice were sacrificed seven days postexposure to crystalline silica.

### 2.3. Cell Coculture

CD19^+^ B cells were immediately isolated from the three groups after the mice were sacrificed by using magnetic-activated cell sorting (MACS) technology (Miltenyi Biotech, Auburn, CA). CD4^+^ T cells were first purified from control group mouse spleen by MACS, and then CD4^+^CD25^+^ Treg and CD4^+^CD25^−^ Teff were separately isolated by positive selection and negative selection according to a magnetic column-based system Treg/Teff cell isolation kit (Miltenyi Biotech, Auburn, CA).

CD4^+^CD25^+^ T cells (1 × 10^5^) or CD4^+^CD25^−^ T cells (1 × 10^6^) were cultured with CD19^+^ B cells (1 × 10^6^) from three different group of mice separately for 72 h in complete RPMI 1640 medium containing 10% FBS (Biological Industries, Kibbutz Beit-Haemek, Israel), 1% HEPES (Sigma-Aldrich), 100 U penicillin-streptomycin (Invitrogen), and 1 *μ*g/ml anti-CD3/CD28 monoclonal antibodies at 37°C in 5% CO_2_. To block the cell-cell contact, transwell insert (0.4 *μ*m pore size; Costar; Corning; USA) was used during cell culture. CD19^+^ B cells were placed in the lower chamber, and Treg/Teff were in the upper chamber.

### 2.4. Flow Cytometry

For B cell staining, PerCP-Cy5.5-conjugated CD19 (BD Pharmingen) and PE-conjugated anti-IL-10 (BD Pharmingen) mAbs were used. For identification of T cells, PerCP-Cy5.5-conjugated CD4 (BD Pharmingen), PE-conjugated CD25 (Miltenyi Biotech, Auburn, CA), Alexa Fluor 488-conjugated anti-IFN-*γ* (BD Pharmingen), PE-conjugated anti-IL-17A (BD Pharmingen), and Alexa Fluor 647-conjugated anti-Foxp3 (BD Pharmingen) were used. Cells from purified B cells of three different groups or the cocultured cells were stimulated with a leukocyte activation cocktail (BD Pharmingen, San Jose, CA, USA) for 5 h, followed by blocking with purified rat anti-mouse CD16/32 (BD Pharmingen) for 10 mins at 4°C. For surface staining, cells were stained with CD19-PerCP-Cy5.5, CD-25-PE, or CD4-PerCP-Cy5.5 mAbs. Cells were washed, fixed, permeabilized, and stained for detection of intracellular cytokines or transcription factors with IL-10-PE, IFN-*γ*-Alexa Fluor 488, IL-17A-PE, or Foxp3-Alexa Fluor 647 mAbs. Cells were subsequently analyzed using a FACSCanto II flow cytometer (BD Biosciences, Franklin Lakes, NJ). Dead cells and crystalline silica particles were gated out according to forward scattering (FSC) and side scattering (SSC). Cells were analyzed with FlowJo Software (TreeStar).

### 2.5. RNA Extraction and Real-Time PCR

Total RNA was extracted from cocultured cells using TRIzol reagent (Invitrogen, Carlsbad, CA, USA), according to the manufacturer's protocol. The RNA concentration and the A260/A280 ratio were determined using a UV spectrophotometer.

Primers were designed with Primer3 (http://bioinfo.ut.ee/primer3-0.4.0/primer3/), and the sequences were checked by performing a BLAST search (http://blast.ncbi.nlm.nih.gov/Blast.cgi) (Table 1). In general, 2 *μ*g total RNA was reverse transcribed in a 20 *μ*l reaction using the following programs: 37°C for 15 min and 85°C for 5 s using PrimeScript RT kit (DRR047A, Takara, Japan). Then, 1 *μ*l of cDNA was used for real-time PCR analysis in a 13 *μ*l reaction volume using Premix Ex Taq II RT-PCR kit (DRR081A; Takara). Amplification efficiency differences between target genes and housekeeping genes were identified by comparing the slopes of the standard curves. The PCR reactions were run on an ABI 7500 cycler (Applied Biosystems) using the following programs: 95°C for 30 s, 40 cycles of 95°C for 5 s, and 60°C for 34 s. Analyses were performed using the 7500 system software (Applied Biosystems).

### 2.6. Cytokine Analysis

Secreted protein levels in the supernatant of cocultured system were examined by CBA assay using mouse Th1/Th2/Th17 cytokine kit (BD Pharmingen) following the manufacturer's instructions. Generally, multiple capture beads were mixed together, including TNF-*α*, IL-6, IFN-*γ*, IL-2, IL-17, and IL-10. The mixed capture beads were cocultured with 50 *μ*l supernatant and detection reagent for 2 h. Beads were washed carefully and resuspended. Samples were analyzed using a FACSCanto II system (BD Biosciences). Data was analyzed with FCAP Array software.

### 2.7. Statistics

Values for all measurements were expressed as the mean ± SEM. Data was analyzed for significance in SPSS software, version 19.0 (SPSS Inc., Chicago, IL, USA) by one-way ANOVA with Student-Newman-Keuls test as specified. All experiments were repeated at least once with consistent results. *P* < 0.05 was considered statistically significant.

## 3. Results

### 3.1. B10 Insufficiency Elevated the Levels of Proinflammatory Cytokines in the Teff and B Cell Coculture System In Vitro

**Figure 1:**
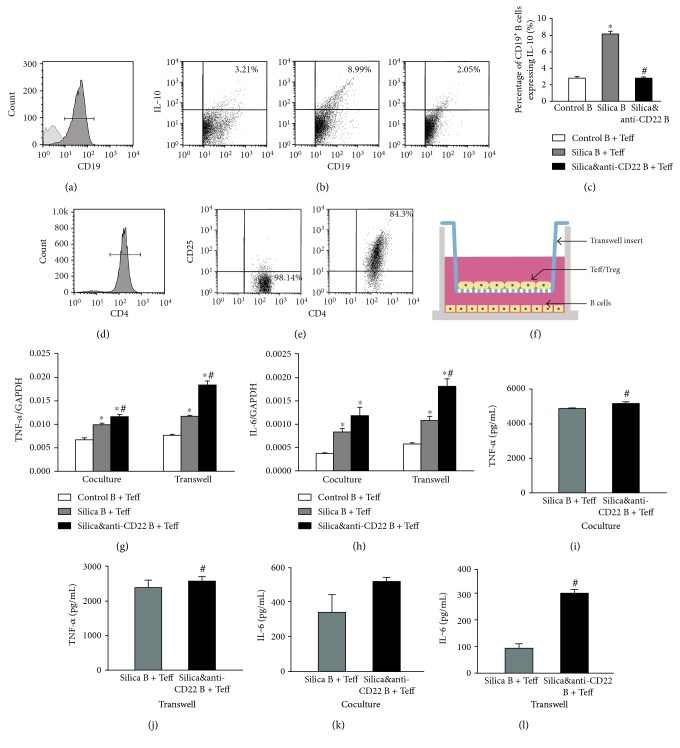
Insufficient B10 promoted the proinflammatory cytokines in the Teff and B cell coculture system in vitro. (a1) CD19^+^ B cells were purified and checked by flow cytometry. (a2, a3) The percentages of B10 in different groups were tested by flow cytometry. (b1, b2) The CD4^+^CD25^−^ Teff and CD4^+^CD25^+^ Treg were purified and checked by flow cytometry. (c) A schematic of B cell and Teff coculture system with insert plates in vitro. (d, e) The expressions of TNF-*α* and IL-6 in different groups of the B cell and Teff coculture system with or without insert plates were assayed by real-time PCR. (f, h) The secretions of TNF-*α* and IL-6 in supernatant of the B cell and Teff coculture system were assayed by CBA. (g, i) The concentrations of TNF-*α* and IL-6 in supernatant of the B cell and Teff coculture system with insert plates were assayed by CBA. (Data was presented as mean ± SEM (*n* = 5). ^∗^*p* < 0.05, significantly different compared with the control B + Teff group. ^#^*p* < 0.05, significantly different compared with the silica B + Teff group.)

CD19^+^ B cells were purified from mouse spleens in different groups using MACS kits as described in the Materials and Methods section. Control B referred to B cells sorted from the control group mice; silica B referred to B cells isolated from the silica group mice; and silica&anti-CD22 B referred to B cells purified from the silica&anti-CD22 group mice treated with anti-CD22 mAb which were also exposed to crystalline silica particles. The purification of CD19^+^ B cell was more than 90% after MACS sorting (Figure 1(a1)). The percentage of B10 in the silica B was obviously higher than that in the control B. Cells from the silica&anti-CD22 B contained less B10 compared with those from the silica B, which was consistent with our previous report [31]. And this difference among the three groups kept significant even after 72 h culture (Figure 1(a2)). Flow cytometry analyses showed the percentages of CD19^+^ IL-10^+^ B10 in different groups (Figure 1(a3)).

Purified control B, silica B, or silica&anti-CD22 B cells were separately cocultured with sorted CD4^+^CD25^−^ Teff cells for 72 h (Figures 1(b1) and 1(b2)). To check the potential role of B10 in modulating inflammatory response, two characterized proinflammatory cytokines TNF-*α* and IL-6 were tested by real-time PCR and CBA. The expressions of TNF-*α* and IL-6 in the silica&anti-CD22 B + Teff group were higher than those in the silica B + Teff group (Figures 1(d) and 1(e)). And this difference was confirmed by CBA, which indicated that insufficient B10 led to elevations of inflammatory cytokines (Figures 1(f) and 1(h)). Then, purified B cells from different groups were cocultured separately with sorted CD4^+^CD25^−^ Teff cells for 72 h with insert plates (Figure 1(c)). The levels of TNF-*α* and IL-6 in culture supernatant of the silica&anti-CD22 B + Teff group also increased compared with the silica B + Teff group (Figures 1(d), 1(e), 1(f), 1(g), 1(h), and 1(i)), which indicated that the role of B10 on inflammatory cytokines was not dependent upon cell-cell contact.

### 3.2. Insufficient B10 Aggravated Th Responses in a Way Independent upon Cell-Cell Contact

**Figure 2:**
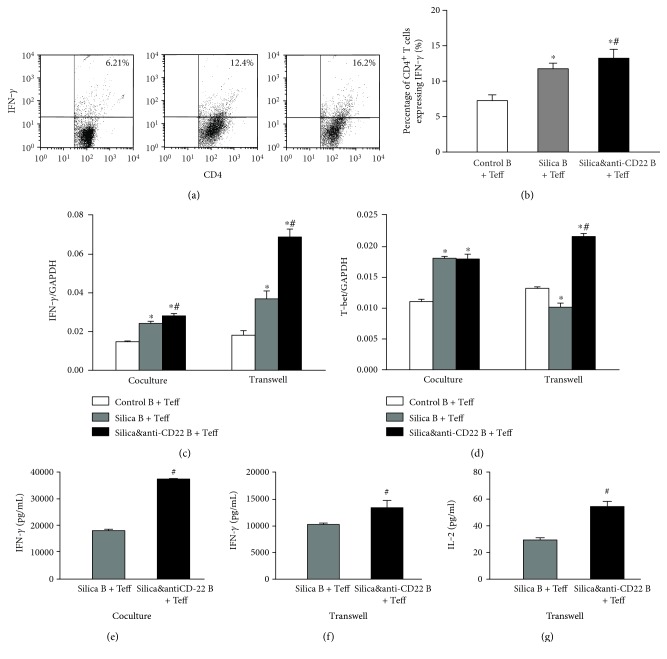
Insufficient B10 aggravated the Th1 response in the Teff and B cell coculture system in vitro. (a1, a2) The percentages of CD4^+^IFN-*γ*^+^ Th1 cells in different groups of the B cell and Teff coculture system were studied by flow cytometry. (b, c) The expressions of IFN-*γ* and T-bet in different groups of the B cell and Teff coculture system with or without insert plates were assayed by real-time PCR. (d) The secretions of IFN-*γ* in supernatant of the B cell and Teff coculture system were assayed by CBA. (e, f) The concentrations of IFN-*γ* and IL-2 in supernatant of the B cell and Teff coculture system with insert plates were assayed by CBA. (Data was presented as mean ± SEM (*n* = 5). ^∗^*p* < 0.05, significantly different compared with the control B + Teff group. ^#^*p* < 0.05, significantly different compared with the silica B + Teff group.)

Multiple Th responses were reported to be involved in crystalline silica-induced lung inflammation in mice. So, we wondered that whether B10 directly affected Th responses in vitro. Flow cytometry analyses showed that the percentage of CD4^+^IFN-*γ*^+^ (Th1) cells in the silica&anti-CD22 B + Teff group increased significantly compared with that in the silica B + Teff group (Figures 2(a1) and 2(a2)). The typical Th1 cytokine, IFN-*γ* [34, 35], was examined by real-time PCR and CBA. The expression and secretion of IFN-*γ* in the silica&anti-CD22 B + Teff group added dramatically compared with those in the silica B + Teff group (Figures 2(b) and 2(d)). Although the difference of Th1 transcript factor (T-bet) [35] between the silica&anti-CD22 B + Teff group and the silica B + Teff group was not significant, Th1 response might be affected by insufficient B10 (Figure 2(c)). The enhanced Th1 response was even notable when coculturing B cells and Teff with insert plates. The elevated level of IFN-*γ* in the silica&anti-CD22 B + Teff group was still significant (Figures 2(b) and 2(e)). The expressions of T-bet and the secretion of IL-2 in the silica&anti-CD22 B + Teff group also enlarged much more than those of the silica B + Teff group (Figures 2(c) and 2(f)).

**Figure 3:**
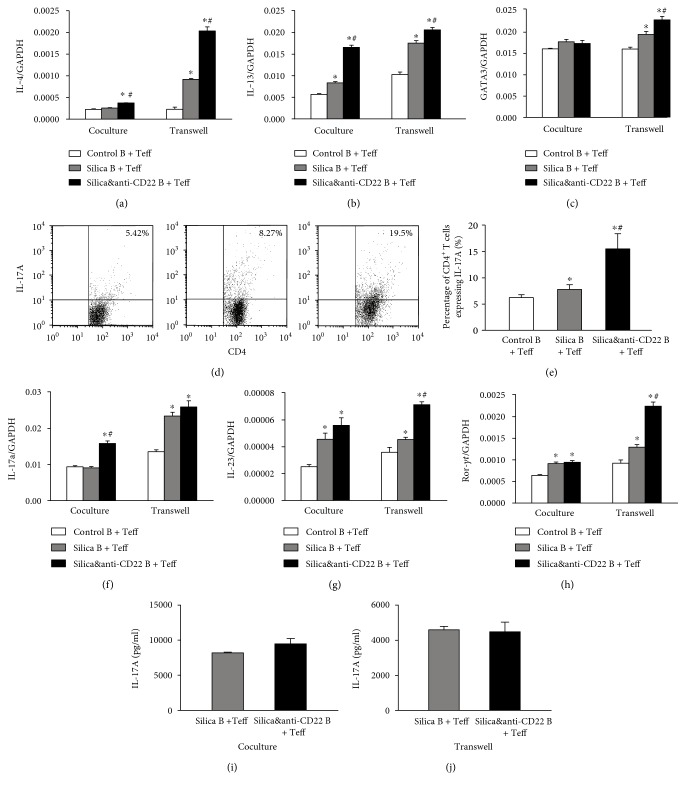
Insufficient B10 promoted the Th2/Th17 responses in the Teff and B coculture system in vitro. (a, b, c) The expressions of IL-4, IL-13, and GATA3 in different groups of the B cell and Teff coculture system with or without insert plates were assayed by real-time PCR. (d1, d2) The percentages of CD4^+^ IL-17A^+^ Th17 cells in different groups of the B cell and Teff coculture system were studied by flow cytometry. (e, f, g) The expressions of IL-17A, IL-23, and ROR-*γ*t in different groups of the B cell and Teff coculture system with or without insert plates were assayed by real-time PCR. (h, i) The secretions of IL-17A in supernatant of the B cell and Teff coculture system with or without insert plates were assayed by CBA. (Data was presented as mean ± SEM (*n* = 5). ^∗^*p* < 0.05, significantly different compared with the control B + Teff group. ^#^*p* < 0.05, significantly different compared with the silica B + Teff group.)

Besides, Th2 response was also reported to play a crucial role in the development of silicosis. In this study, typical Th2 cytokines IL-4 and IL-13 and the Th2 transcript factor (GATA3) [36] were examined in the cocultured system in vitro (Figures 3(a), 3(b), and 3(c)). The expressions of IL-4 and IL-13 both elevated prominently in the silica&anti-CD22 B + Teff group compared with those in the silica B + Teff group (Figures 3(a) and 3(b)). Meanwhile, the increased GATA3 expression clarified the promoted Th2 response as supplement (Figure 3(c)). The effect on Th2 response was not dependent upon cell-cell contact, since the elevated levels of Th2 cytokines and Th2 transcript factor were still significant when coculturing B cells and Teff with insert plates (Figures 3(a), 3(b), and 3(c)).

Another indispensable Th response, Th17, was also demonstrated to be affected by insufficient B10 according to our results. The percentage of CD4^+^ IL-17^+^ (Th17) cells in the silica&anti-CD22 B + Teff group was markedly higher than that of the silica B + Teff group according to flow cytometry (Figures 3(d1) and 3(d2)). The expression and secretion of IL-17A elevated more in the silica&anti-CD22 B + Teff group compared with those in the silica B + Teff group (Figures 3(e) and 3(h)). IL-23 in the silica&anti-CD22 B + Teff group, which had a positive role in promoting the differentiation of Th17 cells, also expressed more than that in the silica B + Teff group (Figure 3(f)). Next, different groups of B cells and Teff were cocultured with insert plates. Although the difference of IL-17A level between the silica B + Teff group and the silica&anti-CD22 B + Teff group was not significant statistically, the expressions of IL-23 and *RORγt* increased dramatically in the silica&anti-CD22 B + Teff group (Figures 3(f) and 3(g)).

### 3.3. B10 Could Enhance Treg Response and Convert Teff into Treg

**Figure 4:**
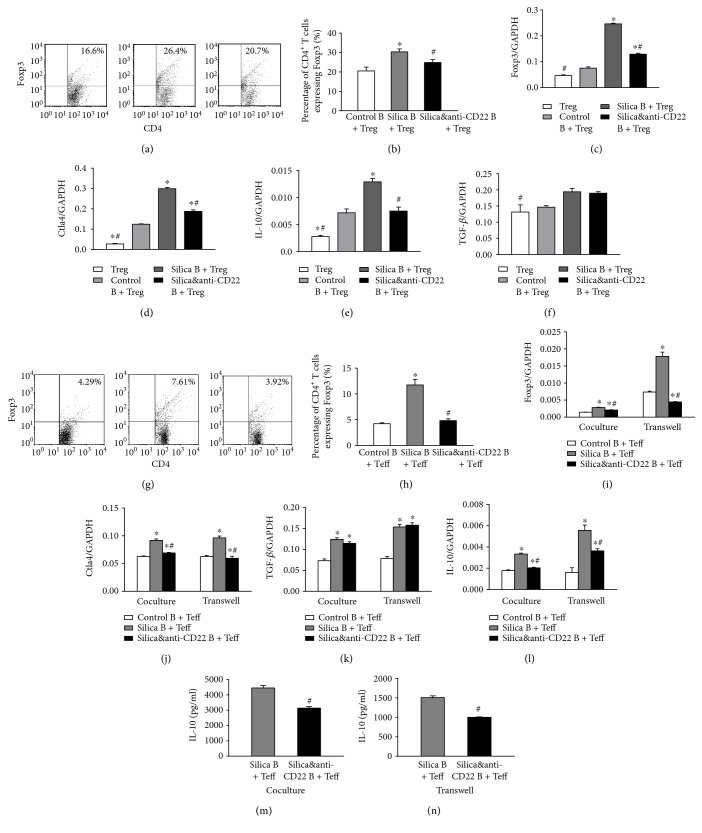
Insufficient B10 both suppressed Treg and inhibited the conversion of Teff into Treg. (a1, a2) The percentages of CD4^+^CD25^+^ Treg in different groups of the B cell and Treg coculture system were studied by flow cytometry. (b–e) The expressions of Foxp3, CTLA4, IL-10, and TGF-*β* in different groups of the B cell and Treg coculture system were assayed by real-time PCR. (f1, f2) The percentages of CD4^+^CD25^+^ Treg in different groups of the B cell and Teff coculture system were studied by flow cytometry. (g–j) The expressions of Foxp3, CTLA4, IL-10, and TGF-*β* in different groups of the B cell and Teff coculture system with or without insert plates were assayed by real-time PCR. (k, l) The secretions of IL-10 in supernatant of the B cell and Teff coculture system were assayed by CBA. (Data was presented as mean ± SEM (*n* = 5). ^∗^*p* < 0.05, significantly different compared with the control B + Teff group. ^#^*p* < 0.05, significantly different compared with the silica B + Teff group.)

CD4^+^CD25^+^ Treg was claimed to have a crucial role in regulating silicosis development and the crystalline silica-induced Th immune response according to our previous study [28, 29]. We firstly cocultured the CD19^+^ B cells and CD4^+^CD25^+^ Treg this time and checked if the change of B10 could influence Treg in vitro. Flow cytometry showed that the percentage of CD4^+^Foxp3^+^ Treg increased obviously in the silica B + Treg group (Figures 4(a1) and 4(a2)). According to real-time PCR assay, the expression of Treg transcript factor (Foxp3) was also suppressed in the silica&anti-CD22 B + Treg group compared with the silica B + Treg group (Figure 4(b)). The expression of Treg functional molecule CTLA4 and the inhibitory cytokine IL-10 also demonstrated the similar trend as Foxp3 (Figures 4(c) and 4(d)). However, the change in another inhibitory cytokine TGF-*β* was not obvious among those groups (Figure 4(e)).

As known, CD4^+^CD25^−^ Teff could be converted into Treg under some circumstances [24, 37, 38]. So, we next detected Treg and its related molecules in the CD19^+^ B cell and CD4^+^CD25^−^ Teff coculture system. The percentage of CD4^+^Foxp3^+^ Treg also enhanced in the silica B + Teff group, which indicated that crystalline silica instillation in mice not only influenced the Treg itself but also promoted the Treg differentiation from Teff. And this differentiation was affected by insufficient B10 based on the fact that the percentage of CD4^+^Foxp3^+^ Treg was restrained in the silica&anti-CD22 B + Teff group (Figures 4(f1) and 4(f2)). The real-time PCR assay on Foxp3 and CTLA4 also supported the demonstration (Figures 4(g) and 4(h)). The situation was also checked in the CD19^+^ B cell and CD4^+^CD25^−^ Teff coculture system with insert plates. And the trend among different groups was not changed when the cell-cell contact was blocked by insert plates (Figures 4(g) and 4(h)). The difference of the expression of TGF-*β* between the silica B + Teff group and the silica&anti-CD22 B + Teff group was not significant in the cocultured system either with or without insert plates (Figure 4(i)). On the other hand, the expression and secretion of IL-10 dramatically decreased in the silica&anti-CD22 B + Teff group compared with those in the silica B + Teff group (Figures 4(j) and 4(k)). And the level of IL-10 was not affected by using insert plates in cocultured system in vitro (Figures 4(j) and 4(k)), which indicated that IL-10 was the major functional cytokine of B10.

## 4. Discussion

**Figure 5:**
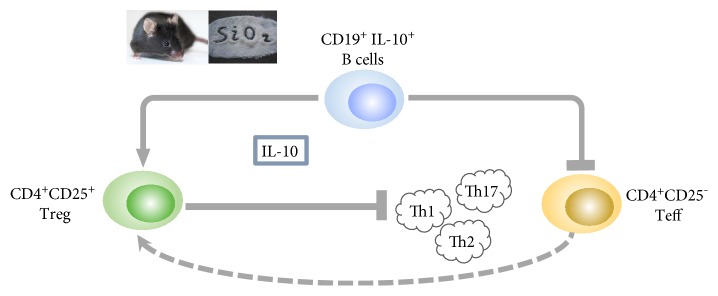
A schematic representation for the regulatory function of B10 on crystalline silica-induced Teff/Treg in vitro.

The molecular mechanisms underlying B10-mediated immune suppression have been studied in various human and murine disease models, such as experimental autoimmune encephalomyelitis (EAE), inflammatory bowel disease (IBD), and rheumatoid arthritis (RA) [13, 39, 40]. Our previous study indicated that B10 could control lung inflammation through modulating Th balance in experimental silicosis in mice [31]. Here, we analyzed the regulatory effects of B10 on the Th responses by using the mouse primary B cell and Teff/Treg coculture system. Our results suggested a multiple capacity of B10 during the crystalline silica-induced inflammation in vitro. First, the insufficient B10 could promote the release of TNF-*α* and IL-6 in the Teff and B cell coculture system in vitro. Second, the lack of B10 was able to directly elevate Th1/Th2/Th17 responses. Third, the deficient B10 could suppress the conversion of Teff into Treg. Fourth, the insufficient B10 was able to restrict the crystalline silica-induced Treg response, which was testified to contribute to the regulation of different Th type responses [28, 29]. This modulated function of B10 was not dependent on cell-cell contact, and in which IL-10 played a crucial role (Figure 5).

These findings, together with our previous study, provide an attractive explanation of how a very small population could maintain a balanced immune environment. In this study, the silica B + Teff group failed to inhibit the secretion of inflammation cytokines compared with the control B + Teff group. It was tempting to speculate that using B cells instead of B10 cells might lead to the result. B cells exhibited a dual role in modulating immune response through either anti-inflammaton or proinflammation in both health and disease. B cells, identified by releasing IL-10, IL-35, and TGF-*β*, had been shown to limit inflammation, autoimmune disease, and tumor, while B cells, identified by secreting TNF-*α*, IL-6, and GM-CSF, had been shown to promote disease [14, 41–44]. So, the release of proinflammation cytokines of B cells might influence the results.

B10 cells were found at low frequencies (1–5%) among spleen B cells in naïve mice but expanded in autoimmunity. They were also found in low numbers within blood, lymph nodes, Peyer's patches, intestinal tissues, and the CNS [20, 32, 45, 46]. It is impracticable to accumulate enough B10 for cell culture. Consequently, we used a B10 depletion strategy to explore its impact. Anti-CD22 mAb, which was reported to eliminate B10 [31–33], was used to restrict the expansion of crystalline silica-induced B10. We found that the frequency of CD19^+^ IL-10^+^ B10 in the silica&anti-CD22 B cells group was much lower than the silica B cells counterparts, even after 72 h culture. This finding was consistent with our previous study; therefore, we used anti-CD22 mAb to investigate the regulatory effect of B10.

Several studies have demonstrated that B10 can inhibit Th1/Th2/Th17 responses and promote CD4^+^CD25^−^ Teff converting into CD4^+^CD25^+^ Treg in autoimmune diseases in vitro [22, 37, 47]. Our results confirmed the function of B10. However, in our previous study, we found that B10 inhibited Th1 responses only. Macrophage plays a crucial role in experimental silicosis [1, 7, 48, 49]. Activation of alveolar macrophages can influence the lymphocytes by secreting cytokines, such as TNF-*α*, IL-6, IL-1*β*, and TGF-*β*. So, the Th2/Th17 cells might be partly affected by alveolar macrophages in vivo. Treg has diverse phenotypes, such as IL-10^+^ Treg, TGF-*β*^+^ Treg, and IL-35^+^ Treg, according to the release of immunosuppressive cytokines [50–52]. In the current study, we found that B10 could convert CD4^+^CD25^−^ Teff into Treg and increase the expression of Foxp3 and CTLA4 as well as the release of IL-10. However, a B-1a cell subset was reported that could induce Foxp3^−^ T cells with regulatory activity which could produce high levels of IFN-*γ* and IL-10, but minimal amounts of IL-4 [53]. IL-10 could convert CD4^+^ T cells into Treg [54]. Our finding was consistent with the clinical study, which B10 could induce Foxp3^+^ T cells that could produce high levels of TGF-*β* and IL-10 with regulatory activity [47].

In correlation with our original hypothesis, B10 could promote the activity of Treg. We cultured the CD19^+^ B cells from the different groups and CD4^+^CD25^+^ Treg from the control group. Foxp3 and CTLA4 have been identified as activated markers of Treg [50–52]. The percentage of CD4^+^Foxp3^+^ Treg and the expressions of CTLA4 and IL-10 increased obviously in the silica B + Treg group. Our findings were similar to the previous studies which detected the effect of B10 exerted on Treg [38, 47, 55, 56].

B10 might modulate Th responses through the direct contact between B10 and CD4^+^ T cells by costimulatory molecules, such as PD-L1, GITR, Tim-1, or CD86 [22, 38, 47, 57, 58]. In the present study, a transwell was used to block the cell-cell contact in vitro. The results showed that the insufficient B10 led to the expansion of Th1/Th2/Th17 responses in cells both cocultured with and without a transwell. The regulatory effect of B10 by secreting IL-10 has been widely discussed [12, 23, 59]. In the current study, the level of IL-10 was much higher in the silica B + Teff group compared with that in the silica&anti-CD22 B + Teff group. However, the level of TGF-*β* was not significantly different between the silica B + Teff group and silica&anti-CD22 B + Teff group. It is tempting to speculate that B10 might mainly suppress the Th responses by releasing IL-10 in experimental silicosis.

## 5. Conclusions

Taken together, our results demonstrated that B10 could suppress crystalline silica-activated proinflammatory cytokines in cocultured system in vitro. The proinhibitory function of B10 on Th responses was independent upon cell-cell contact. B10 could both affect Treg and promote the conversion of CD4^+^CD25^−^ Teff into Treg, which could subsequently suppress the Th responses.

## Figures and Tables

**Table 1:** Primer sequences for real-time PCR.

*Mus musculus* gene name	Forward 5′-3′	Reverse 5′-3′
T-box 21	TCAACCAGCACCAGACAGAGA	TCCACCAAGACCACATCCAC
Interferon gamma	AAGCGTCATTGAATCACACCTG	TGACCTCAAACTTGGCAATACTC
GATA binding protein 3	GGATGTAAGTCGAGGCCCAAG	ATTGCAAAGGTAGTGCCCGGTA
Interleukin 4	ACGGAGATGGATGTGCCAAAC	AGCACCTTGGAAGCCCTACAGA
Interleukin 13	CCCCTGTGCAACGGCAGCAT	GAAGGGGCCGTGGCGAAACA
Tumor necrosis factor-*α*	ACTCCAGGCGGTGCCTATGT	GTGAGGGTCTGGGCCATAGAA
Interleukin 6	CAACGATGATGCACTTGCAGA	CTCCAGGTAGCTATGGTACTCCAGA
RAR-related orphan receptor gamma	ACGGCCCTGGTTCTCATCA	CCAAATTGTATTGCAGATGTTCCAC
Interleukin 17A	GCAAAAGTGAGCTCCAGAAGG	TCTTCATTGCGGTGGAGAGTC
Interleukin 23	ACATGCACCAGCGGGACATA	CTTTGAAGATGTCAGAGTCAAGCAG
Forkhead box P3	CCCATCCCCAGGAGTCTTG	ACCATGACTAGGGGCACTGTA
Interleukin 10	GGGGCCAGTACAGCCGGGAA	CTGGCTGAAGGCAGTCCGCA
Transforming growth factor beta 1	TGTGGAACTCTACCAGAAATATAGC	GAAAGCCCTGTATTCCGTCTC
Glyceraldehyde-3-phosphate dehydrogenase	CAATGTGTCCGTCGTGGATCT	GTCCTCAGTGTAGCCCAAGATG

## Abbreviations

IL-4: Interleukin 4
IL-13: Interleukin 13
IL-10: Interleukin 10
IL-6: Interleukin 6
IL-17A: Interleukin 17A
TNF-*α*: Tumor necrosis factor alpha
IFN-*γ*: Interferon gamma
TGF-*β*: Transforming growth factor beta
IL-23: Interleukin 23
Treg: Regulatory T cells
Teff: Effector T cells
T-bet: T cell-specific T-box transcription factor
GATA-3: GATA binding protein 3
Foxp3: Forkhead box P3
ROR-*γ*t: RAR-related orphan receptor gamma
Th1: T helper type 1 cells
Th2: T helper type 2 cells
Th17: T helper type 17 cells
CD4: Cluster of differentiation 4 receptors
CD19: Cluster of differentiation 19 receptors
GAPDH: Glyceraldehyde-3-phosphate dehydrogenase.
